# Prevalence of Astroviruses in Different Animal Species in Poland

**DOI:** 10.3390/v16010080

**Published:** 2024-01-04

**Authors:** Konrad Kuczera, Anna Orłowska, Marcin Smreczak, Maciej Frant, Paweł Trębas, Jerzy Rola

**Affiliations:** 1 Voivodship Veterinary Inspectorate Katowice, ul. Brynowska 25a, 40-585 Katowice, Poland; konrad.kuczera@katowice.wiw.gov.pl; 2 Department of Virology, National Veterinary Research Institute, 24-100 Puławy, Poland; p.trebas@piwet.pulawy.pl (P.T.); jrola@piwet.pulawy.pl (J.R.); 3 Department of Swine Diseases, National Veterinary Research Institute, 24-100 Puławy, Poland; maciej.frant@piwet.pulawy.pl

**Keywords:** astroviruses, wildlife, companion animals, pigs, prevalence, phylogenetic relationships

## Abstract

Astroviruses (AstVs) are small RNA viruses characterized by a high mutation rate, the ability to recombine, and interspecies transmission, which allows them to infect a multitude of hosts including humans, companion animals, and farmed animals as well as wildlife. AstVs are stable in the environment, and their transmission is usually through the fecal–oral route or via contaminated water and food. Although direct zoonotic transmission was not confirmed, interspecies transmission events have occurred or have been indicated to occur in the past between wild and domestic animals and humans. They cause large economic losses, mainly in the poultry sector, due to gastroenteritis and mortality. In young children, they are the second most common cause of diarrhea. This study involved 166 intestine samples and pools of spleen, lymph node, and kidney samples collected from 352 wild animals, 52 pigs, and 31 companion animals. Astroviruses were detected in the intestine samples and were separately detected in pools of tissue samples prepared for individual animals using a heminested RT-PCR protocol. Amplicons were subjected to Sanger sequencing, and a phylogenetic analysis of 320 nt RNA-dependent RNA polymerase (RdRp) fragments referring to known nt sequences of astroviruses was performed. Astroviral RNA was detected in the intestine samples and/or tissue pools of red foxes (nine positive intestines and six positive tissue pools), rats (two positive intestines and three positive tissue pools), a cat (one AstV detected in an intestine sample), pigs (eight positive tissue pools), and wild boars (two positive pools of spleens, kidneys, and lymph nodes). No astroviral RNA was detected in wild mustelids, dogs, or other small wild animals including rodents. A phylogenetic analysis revealed that the astroviruses detected during this study were mostly host-specific, such as porcine, canine, and rat astroviruses that were highly homologous to the sequences of reference strains. In one of two wild boars, an AstV distinct to porcine species was found with the highest nt identity to *Avastroviruses*, i.e., turkey astroviruses, which suggests potential cross-species transmission of the virus, as previously described. Here, we present the first detection of astroviruses in the population of wild animals, companion animals, and pigs in Poland, confirming that astroviruses are frequent pathogens circulating in animals in the field. Our study also suggests potential cross-species transmission of *Avaastrovirus* to wild boars; however, further molecular characterization is needed.

## 1. Introduction

Wild animals are a source and a reservoir of pathogens, many of which are characterized by their capacity for interspecies transmission and their ability to infect other animal species and humans. Recent papers concerning studies on the prevalence of pathogens in wildlife have demonstrated numerous viruses from viral families including *Adenoviridae*, *Astroviridae*, *Bunyaviridae*, *Circoviridae*, *Coronaviridae*, *Hepadnaviridae*, *Hepeviridae*, *Herpesviridae*, *Paramyxoviridae*, *Polyomaviridae*, *Reoviridae*, *Retroviridae*, *Caliciviridae*, *Circoviridae*, *Rhabdoviridae*, *Flaviviridae*, *Picornaviridae*, *Orthomyxoviridae*, and *Parvovirinae* [1]. Due to the close proximity of some wild animals to humans and the zoonotic potential of the pathogens they harbor, wild animals might pose a threat to public health.

Astroviruses are small RNA viruses characterized by a high mutation rate, the ability to recombine, and interspecies transmission, which allows them to infect a multitude of hosts [2,3]. They have been detected in over 80 host species, including humans, bats, companion animals (dogs and cats), and livestock (pigs, chickens, and cows), as well as wild animals such as wild boars and rats. Farmed animals like turkeys and mink are particularly affected by astroviruses [4]. AstVs cause diseases that range from asymptomatic to systemic. Human astroviruses are most commonly associated with diarrhea and gastrointestinal symptoms in the elderly and immunocompromised people and are the second most common cause of gastroenteritis in young children, after rotavirus [4,5]. In farmed mink infected with astrovirus type 1 (MAstV-1), neurological symptoms were reported [6], which caused shaking mink syndrome (SMS), preweaning mink diarrhea (PMD), and wet mink syndrome (WMS). Encephalopathies caused by astrovirus infections have also been detected in an immunosuppressed boy, cattle, sheep, pigs, and alpacas [7,8,9,10]. The largest economic losses, however, are reported in the poultry sector, mainly due to severe gastroenteric illnesses and mortality.

A given host species can often be infected by distinct astrovirus strains that are reported for bats, turkeys, domestic pigs, sea lions, and domestic sheep [4]. This means that the astroviruses infecting one host species can be highly diverse, with different lineages reflecting independent origins. The ability to recombine, high mutation rate, RNA genome, and interspecies transmission mean that astroviruses are highly genetically diverse, evolve rapidly, and are able to quickly adapt to novel niches. While direct zoonotic infection of humans by AstVs was not demonstrated, humans in close contact with a cohort of turkeys tested positive for turkey astrovirus-2 [11]. Using phylogenetic tools, a few studies revealed that novel human astroviruses were phylogenetically clustered more closely with animal astroviruses (mink, ovine, and rat) than with human AstVs [12]. Another example of suspected cross-species astrovirus transmission was demonstrated from chicken or turkey astrovirus into mink [13]. The identification of animal astroviruses in human clinical and sewage samples [14] suggests that humans might be exposed to animal astrovirus strains and that the zoonotic interspecies transmission of astroviruses is possible. The most common route of exposure is the fecal–oral route. Water used for human recreation or drinking could be contaminated by the agricultural runoff of infected wild animals, whereas pigs and wild boars could become directly infected through the ingestion of feces from another infected species [15].

Despite astroviruses being clinically and agriculturally significant and a potential public health concern, they are the least studied enteric RNA viruses [2]. Here, we present data concerning a study of wild animals (red foxes; wild boars; and other small wildlife such as racoons, martens, and rats), companion animals (dogs and cats), and farm animals (pigs) to provide an update on the prevalence of astroviruses in the field, the phylogeography and distribution of distinct astrovirus strains in the wildlife in terms of the potential emergence of novel human and animal astroviruses, and potential zoonotic sources of astroviruses for humans. Simultaneously, this is the first report on astrovirus prevalence in foxes and small wild animals like racoons, martens, and rats in Poland. Studies on astrovirus screening in animals in Poland were previously performed only in bats found dead and collected during passive rabies surveillance and in farmed animals like poultry and mink [16,17,18].

## 2. Materials and Methods

### 2.1. Samples

Wild boar and pig tissue samples were collected during African Swine Fever monitoring, whereas the remaining animal tissue samples were sent by regional laboratories for rabies in Poland. These samples had tested as non-rabid during rabies surveillance. All of the swine samples were evaluated to be ASFV-negative, although 3 pig samples were collected from a farm where 30 pigs died suddenly one night. During the necropsy of the pigs, petechiae on the skin of the abdomen and auricles were reported and the lungs appeared bloodied but the spleen was unchanged, suggesting ASFV infection. Clinical post-mortem examinations, however, failed to determine the cause of the animals’ sudden demise. The remaining swine samples were collected from clinically healthy animals during routine monitoring. Some wild boars were found dead as victims of traffic accidents.

**Figure 1:**
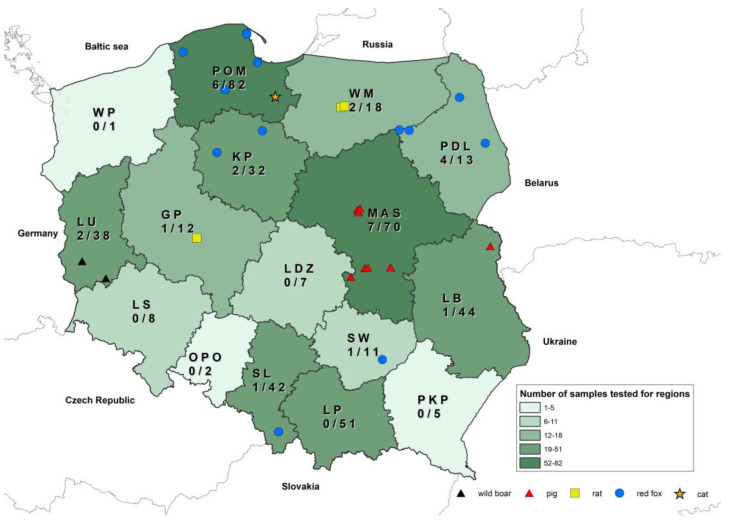
Distribution of astroviruses detected in this study with numbers tested as denominators.

This study involved 166 intestine samples from the 435 animals included in the study. Intestines were collected from red foxes (*n* = 57), cats (*n* = 2), raccoons (*n* = 18), martens (*n* = 18), squirrels (*n* = 18), wild mink (visons, *n* = 13), rats (*n* = 7), dogs (*n* = 5), badgers (*n* = 3), raccoon dogs (*n* = 2), hedgehogs (*n* = 1), a polecat (*n* = 1), a grub (*n* = 1), and an ermine (*n* = 1). Pools of tissue samples of spleens, lymph nodes, and kidneys were collected from 352 wild animals, including red foxes (*n* = 144), wild boars (*n* = 71), raccoons (*n* = 34), raccoon dogs (*n* = 9), martens (*n* = 25), otters (*n* = 3), squirrels (*n* = 20), badgers (*n* = 12), wild American mink (visons) (*n* = 12), rats (*n* = 9), hedgehogs (*n* = 2), mink (*n* = 3), a grub (*n* = 1), a ferret (*n* = 1), a polecat (*n* = 1), a roe deer (*n* = 1), a muskrat (*n* = 1), an ermine (*n* = 1), a wolf (*n* = 1), and a wild hamster (*n* = 1). In total, 31 pools were collected from companion animals (cats (*n* = 27) and dogs (*n* = 4)), and 52 tissue samples were collected from swine. The tested samples of animals originated from the entire area of Poland. The highest numbers of samples were collected in Mazowieckie (MAS) and Pomorskie (PM) voivodeships, whereas the lowest numbers were delivered from Zachodniopomorskie (WP), Opolskie (OPO), and Podkarpackie (PKP) voivodeships (Figure 1).

### 2.2. Molecular Detection of AstVs and Sequencing

For the molecular characterization, total RNA was extracted from pools of 10% (*w*/*v*) homogenates of kidneys, spleens, and lymph nodes or 10% homogenates of individual intestines. RNA was extracted automatically using an IndiMaq Pathogen Kit (Indical Biosciences Gmbh, Leipzig, Germany) and an automated processor of viral RNA/DNA extraction with a magnetic particle (Indical). Nucleic acids were eluted in 100 μL of AVE buffer and immediately subjected to AstV detection or kept frozen at −80 °C. The presence of astroviral RNA was detected using the method previously described by Chu et al. [19].

Positive amplicons were detected via separation in 1.5% agarose gel, and after purification they were subjected to Sanger sequencing in both directions in the commercial service Genomed Sp. z o.o. (Warsaw, Poland).

### 2.3. Phylogenetic Analysis

The nucleotide sequences of the 320 bp RdRp gene of AstVs detected during this study were aligned using a Clustal W Multiple alignment, and a phylogenetic neighbor-joining tree was generated using an appropriate evolutionary model bootstrapped on a set of 1000 replicates with Mega 5 software v. 5110222 [20]. Some reference nt sequences, particularly TAstVs, were shorter; therefore, the similarity matrix of the TAstV detected in a wild boar during this study was carried out for 230 nt of the RdRp fragment. The similarity matrix was created using BLOSUM62 in BioEdit software v. 7.0.5.3.

## 3. Results

Astroviral RNA was detected in 12 out of 166 tested intestines (6.7%) collected from nine red foxes (13.6%), two rats (22.2%), and one cat (3.6%). Additionally, astroviruses were detected in 19 pools out of 435 tissue samples prepared for individual animals collected from pigs (*n positives* = 8; 15.4% of tested swine), wild boars (*n positives* = 2; 2.8% of tested wild boars), red foxes (*n positives* = 6; 4.4% of tested red foxes), and rats *(n positives* = 3; 33.3% of tested rats). In all animals where astroviruses were detected in the intestines, AstVs were also found in the pools of homogenates from the kidneys, spleens, and lymph nodes. For animals that were not tested due to a lack of intestines, positive pools of organs were also detected in two wild boars, eight pigs, and one rat. For all positive amplicons, Sanger sequencing and further phylogenetic analysis confirmed the detection of distinct astroviruses. No astroviral RNA was detected in the remaining wild and companion animal tissue samples despite testing a large number of samples within animal species like mustelids (*n* = 80 of total). The highest percentage of positive results for intestines and/or pools of tissue samples was found in rat samples collected in October 2021 in Warminsko-Mazurskie (WM: *n* = 2) and Wielkopolskie (GP: *n* = 1) voivodeships. However, the number of rats tested was low, i.e., only nine rats, due to the low number of rats collected for passive rabies surveillance. In red foxes, astroviruses were detected in nine intestines and six pools of tissue samples from five red foxes collected in Pomorskie (PM) voivodeship between March and August 2022, four red foxes sampled in Podlaskie (PDL) voivodship in July 2021, two red foxes sampled in Kujawsko-Pomorskie (KP) voivodship between November 2021 and April 2022, and two red foxes collected in Świętokrzyskie (SW) voivodeship in August 2022 and Śląskie (SL) voivodeship in June 2022.

No intestines were collected from pigs or wild boars due to the fact that swine samples were gathered during ASF monitoring, for which intestines are not applicable. However, astroviruses were detected in 15.5% of tested pools of pig tissues (8 positive pools of spleens, kidneys, and lymph nodes out of 52 tested) collected at three farms located in Mazowieckie voivodeship (MAS) and one farm located in Lubelskie voivodeship (LB) between April and July 2021. Only two astroviruses were detected in pools of kidneys, spleens, and lymph nodes from wild boars collected in Lubuskie voivodeship between May and June 2021. All details concerning the prevalence of astroviruses are presented in Table 1.

The astroviruses detected in red foxes revealed the strongest phylogenetic relationships with canine reference astroviruses isolated from red foxes and wolves in Italy and dogs in China, while AstVs detected in Polish rats showed the highest similarity with rat astroviruses collected in Europe (Germany and Hungary), China, and Russia. The astroviruses detected in pigs in Poland were clustered in two previously known groups of porcine AstVs. Four of them belonged to the PAstV-2 group, with the highest nt identity to astroviruses isolated from *Sus scrofa* in Croatia, Slovakia, Canada, and China, and the two remaining AstVs clustered with porcine astrovirus 4 (PAstV-4), revealing the highest percentage of nt identity to AstVs isolated from *Sus scrofa domesticus* in Croatia and Slovakia. The astrovirus detected in a cat’s intestine revealed the highest nt sequence identity to feline astrovirus using a BLAST bioinformatic tool.

**Figure 2:**
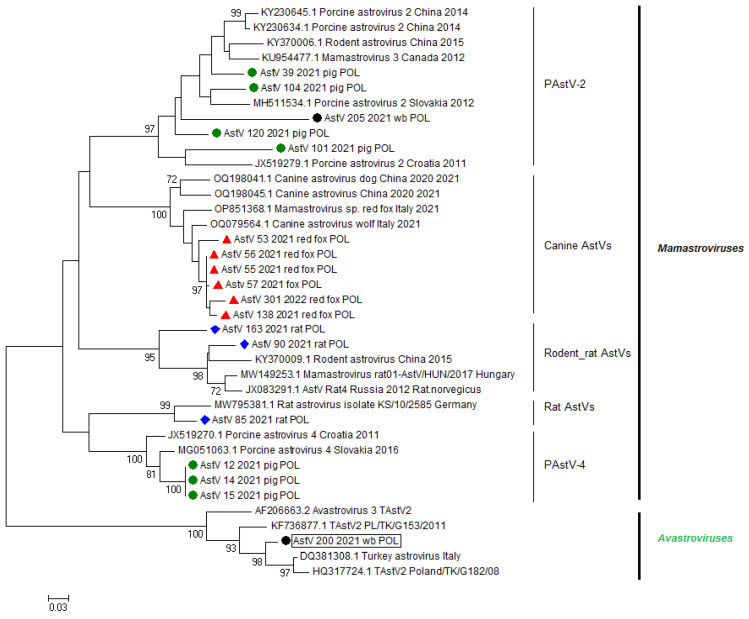
Phylogenetic relationship of nt sequences of astroviruses detected during this study with reference nt sequences available in GenBank database based on 320 bp fragment of RdRp gene. Phylogenetic tree was generated using the neighbor-joining method (Kimura2 parameter) as implemented in Mega5 software. Bootstrap values (1000 replicates) over 70%, indicating significant support for the tree topology, are shown next to the branches. All nt sequences of astroviruses detected during this study are highlighted with indicators: ●—porcine astroviruses, ▲—astroviruses detected in red foxes, ♦—astroviruses detected in rats, ●—included in a black box indicates *Avastrovirus* detected in wild boar.

**Figure 3:**
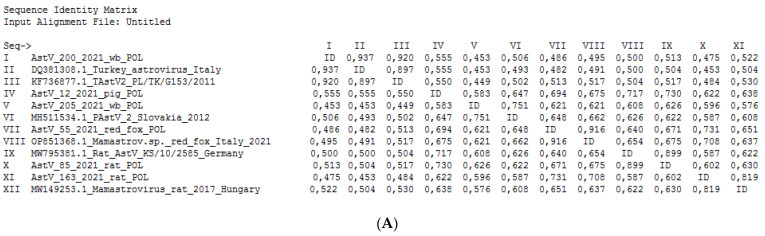

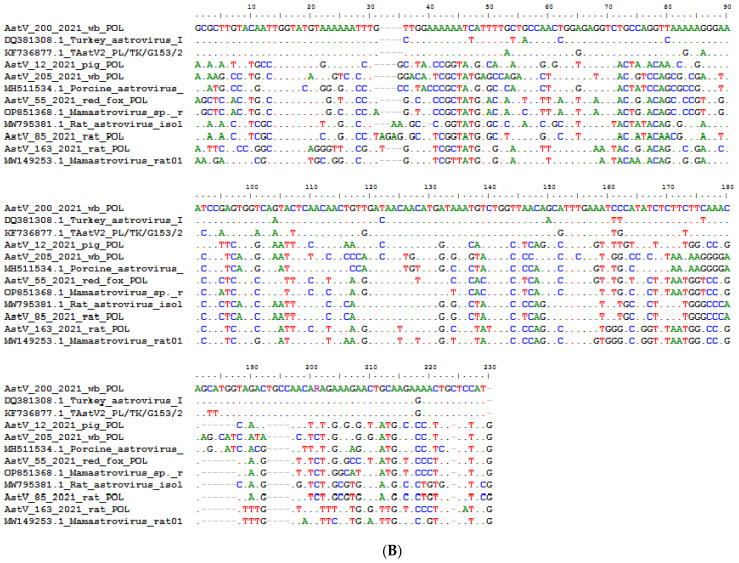
Percentage of nt identity (**A**) and the alignment (**B**) of 230 bp RdRp fragments of representative strains of different astrovirus species used in phylogenetic analysis with TAstV detected in wild boar (AstV_200_2021_wb_POL).

The lowest host specificity was determined for the astroviruses detected in wild boars. One of two astroviruses belonged to the PastV-2 group, revealing the highest percentage of nt identity to AstVs isolated from pigs in Poland (this study) and pigs and brown rats (*Rattus norvegicus*) collected in China. The second astrovirus isolated from a wild boar in this study (AstV_200_2021_wb_POL) was the most homologous to AstVs detected in farmed turkeys and guinea fowl in Italy between 2000 and 2005 (93.7% homologous nt within 230 nt RdRp fragment). The percentage of nt identity between AstV_200_2021_wb_POL and representatives of PAstV ranged between 45.3 and 55.5%. The phylogenetic relationships of AstVs with reference nt sequences of astroviruses are presented in Figure 2, whereas the percentages of nt identity and the alignment of representative strains of different astrovirus species used in the phylogenetic analysis with the TAstV detected in a wild boar are presented Figure 3.

## 4. Discussion

Wild animals represent a large and often poorly studied reservoir of pathogens that play a key role in their transmission to humans and animals and in the emergence of new, previously unknown pathogens. The COVID-19 pandemic has vividly demonstrated the tremendous importance of human–animal contact in the emergence of zoonotic diseases and in particular the role of wild animals as potential reservoirs and hosts of viruses. Therefore, increasing attention has begun to be paid to wild animals as potential sources, reservoirs, and/or vectors of pathogens that can cross interspecies barriers and infect humans and both domestic and wild animals. As a recent example, highly pathogenic avian influenza virus (H5N1) infections have been detected in wild and domestic animals [21,22]. According to “Asia Pacific strategy for emerging diseases: 2010”, around 60% of emerging human infections are estimated to be zoonotic in nature, and among these pathogens more than 70% originate from wildlife species [23].

Astroviruses mostly infect subclinically [24], but in immunocompromised patients, children, and elderly patients, they can lead to death due to diarrhea and gastrointestinal disorders [25]. Astroviruses have also been associated with respiratory illness and encephalitis [10,26]. Several strains of *Astroviridae* have been shown to be pathogenic in animals, suggesting they may be major causative agents of enteric disease, like in poultry, in which infections with astroviruses are more severe than in mammals, sometimes leading to the deaths of birds and significant economic losses. In mink and bovine species in particular, neurological symptoms were observed. Studies on the genetic evolution of astroviruses indicate that it is possible to cross the interspecies barrier and transmit astroviruses from pigs to cats and then from cats to humans. Global environmental and social changes can alter species–species interactions and increase exposure to viruses with zoonotic potential. Astroviruses, which lack a fragile lipid envelope, are stable for long periods in the environment [27]. They are common water contaminants, and transmission is thought to occur via the fecal–oral route [28]. Because of their high environmental stability and wide prevalence in different hosts, opportunities for spillover are abundant. The identification of animal astroviruses in human clinical and sewage samples suggests that humans may be exposed to animal astroviruses. Despite astroviruses being highly clinically and agriculturally significant, they are poorly studied so far. Therefore, the surveillance of astroviruses in wildlife hosts is likely to enhance the knowledge of circulating astroviruses and disease emergence potential prior to spillover to livestock and companion animals as well as, potentially, to people. Here, we provide more data on the prevalence of AstVs in different animal species with an emphasis on wild animals as an underestimated source of different pathogens.

Previous studies on AstVs in farmed and companion animals have demonstrated that turkeys, ducks, chickens, guinea fowl, pigeons, geese, mink, sheep, cattle, pigs, and cats are hosts of astroviruses [3,16,17]. In the population of wild animals, AstVs were mostly detected in bat samples, with several studies performed in wild boars in Slovakia and Hungary [29,30]. Metagenomics studies performed on rodents in China and red foxes in the Netherlands revealed novel rodent and fox astroviruses, respectively [31,32]. In our study, we screened a broad range of animal species using the method Chu et al. [19] successfully applied for the detection of astroviruses in bats in Poland [18], in wild boars in Slovakia [29], and in different wild animals in Italy [33]. Although astroviruses were most frequently detected in rat samples, with a prevalence of 33.3%, the number of samples tested was only nine due to the low number of rat samples submitted during rabies surveillance. The rat astroviruses detected in Poland revealed high host specificity, reflecting high percentages of nt identity (over 90%) to reference rat astroviruses collected from *Rattus norvegicus* in Germany, Hungary, Russia, and China. Simultaneously, they revealed percentages of nt identity around 64.7–78.4% between rat astroviruses detected in Poland, which might confirm the high astrovirus strain diversity previously observed in small animals, primarily rats and other rodents but also bats, all commonly known to form both species-specific clusters, reflecting endemic transmission, and co-clusters of astrovirus strains with other host species, reflecting widespread interspecies transmission. The rat astroviruses detected in Poland formed a group separate from the astroviruses identified in other mammals in Poland, eliminating interspecies transmission events. Despite the low number of rats tested, our study improves the knowledge of the diversity of rat astroviruses and the role of these animals in astrovirus transmission between various species.

Next, the highest number of positive samples was obtained in pigs, for which a prevalence of 15% was reported. Porcine astroviruses (PAstVs) were detected at three farms located in Mazowieckie voivodship and one in Lubelskie voivodship, and the animals had previously tested as ASFV-negative. Five pigs were clinically healthy, whereas the remaining three were sampled at a pig farm in Mazowieckie voivodeship, where thirty pigs died suddenly one night with clinical and pathological signs suggesting ASFV infection. No diarrhea or respiratory or neurological signs suggesting astroviral infection were observed. The astroviruses detected in pigs in Poland clustered with two lineages (PAstV-2 and PAstV-4) of the five porcine astrovirus lineages (PAstV1-5) recognized so far, including the reference Mamastrovirus 3, first detected in pigs in the U.K. and the USA [34] and now recognized to have a worldwide distribution. The astroviruses detected in pigs in Poland (PAstV-2 and PAstV-4) were revealed to be highly homologous to astroviruses detected in *Sus scrofa* in Croatia, Slovakia, China, and Canada, supporting the theory that astroviruses are widely circulating in swine worldwide. The relatively high prevalence of AstVs in pigs in Poland strictly corresponds to other studies reporting up to 80% astrovirus detection in pigs, which confirms that pigs may be persistently infected with various strains of PAstV [35]. Swine are supposed to be highly permissive to astrovirus infection, often without symptoms of disease, and that was supported by our study. The astroviruses detected in wild boars in turn showed the lowest host specificity and the high diversity of AstVs. One of the astroviruses detected in wild boars was revealed to be highly homologous to PAstV-2 isolated from pigs in Poland (this study), Slovakia, and China, which confirms the hypothesis that astroviruses detected in wild boars may be derived from commonly circulating porcine astrovirus strains [30]. The second astrovirus, AstV_200_2021_wb_POL, which was identified in a wild boar killed on a road in Lubuskie voivodeship, showed the highest degree of genetic similarity to distinct TAstV-2 *Avastroviruses* detected in farmed turkeys and guinea fowl isolated at the beginning of this century in Italy. Astroviruses infecting guinea fowl are closely related to turkey TAstV-2 strains, based on the analysis of the RdRp region; however, the capsid region forms a discrete cluster in a lineage of unassigned viruses, suggesting a closer phylogenetic relation to a chicken astrovirus strain (CAstV-B). Taking into account that the phylogeny in our study was performed for the highly conserved RdRp gene to improve the data of the phylogenetic relationships and common roots of the AstV_200_2021_wb_POL isolate, further studies primarily concerning whole-genome sequencing should be carried out. Chu et al. [36] demonstrated the detection of phylogenetically closed astroviruses in wild doves and pigeons and found astroviruses in wild waterfowl that were genetically related to AstVs that circulated in ducks, chickens, and turkeys, suggesting multiple interspecies transmissions between wild birds and domestic poultry populations. In the past, there were few suggestions of interspecies transmission of Avastroviruses via feed from infected birds to mink in China [13]. Avastrovirus has also been detected in cat feces in Florida, while chicken astroviruses and bat astrovirus were identified in wild boars in Slovakia [29], all without a true origin. In addition, non-human primates were shown to harbor a wide spectrum of mammalian and avian astroviruses [37]. In our study, we identified the avastrovirus TAstV-2 in a wild boar, but the origin of this astrovirus is also unknown. Our observations suggest that the astrovirus detected in a wild boar (AstV_200_2021_wb_POL) was probably transmitted across species via feed from a wild bird or poultry to a wild boar like the previously suggested interspecies transmission events of bat astroviruses via food to wild boars [29]. The canine astroviruses detected in red foxes in Poland represent the first report of astrovirus detection in foxes in Poland and the fourth report in the world following a study conducted in the Netherlands, Australia, and Italy [31,33]. In the Netherlands and Australia, astroviruses were detected using a metagenomics approach, whereas in Italy a broad range of primers designed by Chu et al. [19] were applied, leading to the detection of AstVs in 5 out of 12 tested samples. In the Netherlands, fox astroviruses were mainly identified as two novel astroviruses called F5 and F4, of which F5 was mostly related to the human astrovirus HMO (human, mink, and ovine) and astroviruses detected in mink, swine, and California sea lions. The second novel astrovirus, F4, was most closely related to astroviruses detected in mice, pigs, and wild boars. In our study, fox astroviruses were identified in 6 out of 144 tested red fox samples. All six sequenced astroviruses revealed a high degree of genetic identity to canine astroviruses isolated from foxes and wolves in Italy [33] and from a dog from China collected in 2020/2021, suggesting strict host specificity of astroviruses circulating in canines. Red foxes are distributed across the entire Northern Hemisphere and have a broad habitat, ranging from urban areas and farmland to remote forests; therefore, they may be a source of astrovirus infection for other members of the order Carnivora, particularly companion animals like dogs or cats living close to humans.

AstV was found in one intestine of 27 tested cats with a weakly positive RT-PCR result, enabling Sanger sequencing. No astroviruses were found in representatives of dogs in Poland, although previously astroviruses were detected in dogs with diarrheal disease. However, AstV RNA was also detected in 7 of 75 swabs (9.3%) obtained from asymptomatic young dogs in several European countries, the USA, and China [4,38]. Feline astroviruses were identified in the USA, Australia, New Zealand, Germany, Italy, and England [4,39,40,41]. Cats and dogs harbor astrovirus strains more closely related to human astroviruses than the viruses harbored by many other animal species. In particular, feline astroviruses appear to be closely related to human strains. The lack of astroviruses detected in our study can be explained by the fact that a small number of samples collected from companion animals were tested. Surprisingly, no astroviruses were detected in wild mustelids even though 34 raccoons, 25 martens, 12 visons, and a few other animal species belonging to the mustelids were tested. This suggests that wild mustelids are not a reservoir of astroviruses, but more intensive studies are needed.

All astroviruses detected in our study, but not the *Avaastrovirus* detected in a wild boar, revealed high host specificity. The astroviruses detected in pigs and wild boars were the most homologous with porcine astroviruses detected in Canada or China, AstVs detected in rats clustered with rodent and rat astroviruses detected in Europe and China, and AstVs detected in red foxes were the most homologous with canine astroviruses isolated in Italy and China. High percentages of nt sequence similarity with AstVs isolated in several continents indicate that astroviruses display high host specificity, although they are characterized by high mutation and recombination rates that could generate new variants of astroviruses that are able to infect new hosts. Previous reports on the phylogenetic relationships of astroviruses suggested that *Mamastroviruses*, regardless of the origin of isolation, are host-specific. Viruses isolated from a given animal species in Europe, Asia, the USA, or Australia showed high percentages of nt sequence similarity among themselves. Our study corresponds with that, revealing that the percentages of nt sequence identity for the astroviruses in our study and reference nt sequences ranged between 75.9 and 93.6% for porcine, red fox, and rat samples analyzed within a given animal species. Therefore, this confirms the general statement that the evolutionary history of RNA viruses like astroviruses follows the evolutionary history of their hosts, indicating that cross-species transmission events are the exception, rather than the rule [42]; however, interspecies transmission events cannot be excluded, as they were established multiple times.

## 5. Conclusions

In conclusion, our study, which analyzed a broad range of wild and companion animals as well as pigs, confirmed that astroviruses are a relatively frequent pathogen circulating in the field. This was established for both intestines and extra-intestine samples like pools of spleens, kidneys, and lymph nodes. They are mostly host-specific, such as porcine, canine, and rat astroviruses that are highly homologous to their reference strains. The astroviruses detected during this study in the intestine samples and pools of tissue organs revealed high genetic diversity and clustered in five groups within the *Mamastroviridae* genera, with one isolate belonging to the *Avastroviruses*. A wild boar was found to host an AstV distinct to porcine species with the highest degree of nt identity to turkey astrovirus 2, which suggests a potential cross-species transmission of the virus from turkeys, as was previously supposed.

## Figures and Tables

**Table 1:** The prevalence of AstVs in different animal species included in this study.

Species	Intestines No. of Positives/No. of Samples	Intestines Positive % (95% CI)	Pools of Kidneys, Spleens, and Lymph Nodes No. of Positives/No. of Samples	Pools of Organs Positive % (95% CI)
**wild boar**	**0/0**	**-**	**2/71**	**2.8 (0.5–9.6)**
**red fox**	**9/57**	**13.6 (7.3–23.9)**	**6/144**	**8.2 (1.9–8.8)**
raccoon	0/18	0.0 (0.0–17.6)	0/34	0.0 (0.0–10.2)
raccoon dog	0/2	0.0 (0.0–82.2)	0/9	0.0 (0.0–29.9)
marten	0/18	0.0 (0.0–17.6)	0/25	0.0 (0.0–13.3)
squirrel	0/15	0.0 (0.0–20.4)	0/20	0.0 (0.0–16.1)
badger	0/3	0.0 (0.0–56.1)	0/12	0.0 (0.0–24.2)
**rat**	**2/7**	**22.2 (3.9–54.7)**	**3/9**	**33.3 (12.1–64.6)**
otter	0/1	0.0 (0.0–94.9)	0/3	0.0 (0.0–56.1)
wild American mink (visons)	0/12	0.0 (0.0–24.2)	0/12	0.0 (0.0–24.2)
hedgehog	0/1	0.0 (0.0–94.9)	0/2	0.0 (0.0–56.1)
mink	0/1	0.0 (0.0–94.9)	0/3	0.0 (0.0–56.1)
grub	0/1	0.0 (0.0–94.9)	0/1	0.0 (0.0–94.9)
ferret	0/0	-	0/1	0.0 (0.0–94.9)
polecat	0/1	0.0 (0.0–94.9)	0/1	0.0 (0.0–94.9)
roe deer	0/0	-	0/1	0.0 (0.0–94.9)
muskrat	0/0	-	0/1	0.0 (0.0–94.9)
ermine	0/1	0.0 (0.0–94.9)	0/1	0.0 (0.0–94.9)
wolf	0/0	-	0/1	0.0 (0.0–94.9)
wild hamster	0/0	-	0/1	0.0 (0.0–94.9)
**swine**	**0/0**	**-**	**8/52**	**15.4 (8.0–27.5)**
**cat**	**1/23**	**3.6 (0.2–17.7)**	**0/27**	**0.0(0.0–49.0)**
dog	0/5	0.0 (0.0–43.4)	0/4	0.0 (0.0–12.5)
**Total**	**12/166**	**6.7 (3.9–11.4)**	**19/435**	**4.4 (2.8–6.7)**

Species with positive results were bolded.

## Data Availability

Data are contained within the article.
